# Is the Supreme Court veering rightward? The ebb and flow of representation

**DOI:** 10.1093/pnasnexus/pgag060

**Published:** 2026-03-11

**Authors:** Stephen Jessee, Neil Malhotra, Maya Sen

**Affiliations:** Department of Government, University of Texas, Austin, TX 78712, USA; Graduate School of Business, Stanford University, Stanford, CA 94305, USA; John F. Kennedy School of Government, Harvard University, Cambridge, MA 02138, USA

**Keywords:** Supreme Court, representation, ideology, public opinion

## Abstract

Conducting novel surveys that allow the first direct comparisons between Supreme Court decisions and public preferences, Jessee et al. find that the Court moved sharply to the right between 2020 and 2021 and attribute this change to the replacement of Justice Ruth Bader Ginsburg with Justice Amy Coney Barrett. We extend Jessee et al.'s analysis by presenting additional data gathered between 2022 and 2025. We find that the Supreme Court maintained its conservative position in 2022 but then moderated in 2023 following the backlash to the decision in Dobbs v. Mississippi (2022), which repealed Roe v. Wade (1973). We show that despite the composition of the Court remaining stable and the identity of the median voter being unchanged between 2021 and 2025, there is an ebb and flow to the representativeness of Court decisions, with the institution sometimes further to the right of the public and then sometimes shifting closer to the average voter. However, despite these important periodic shifts, the Court has, since 2021, generally remained in a more conservative position relative to the ideological positioning of the American electorate. Our findings have important implications for the legitimacy of the Court and the stability of the rule of law.

## Introduction

Are the decisions of the Supreme Court out of step with the public? Addressing this question is of scholarly importance given the pivotal role the Court plays in making policies that affect the everyday lives of Americans on issues such as abortion, employment discrimination, and affirmative action. Although the Court is not designed to be a responsive, representative institution, previous research has shown that when the Court is ideologically disconnected from the public, it can decrease the institution's perceived legitimacy, incentivize elites to denigrate the Court, and threaten the rule of law (1–3).

In a recent issue of the “Proceedings of the National Academy of Sciences,” Jessee et al. leverage a methodology for directly comparing public opinion against Supreme Court decisions (4), showing that the Supreme Court veered sharply to the right between 2020 and 2021 after liberal Justice Ruth Bader Ginsburg was replaced by conservative Justice Amy Coney Barrett: “the gap between the court and the public has grown since 2020, with the court moving from being quite close to the average American to a position that is more conservative than the majority of Americans” (1). However, their analysis only leverages 2 years of data, meaning it is uncertain whether this 1-year increase in conservatism was a blip or reflects an enduring change in the Court's ideology following the death of Justice Ginsburg.

This study replicates the methodology of Jessee et al. (4) and extends the time series through the 2024–2025 term, increasing the number of waves from two to six. We find that although the Court is estimated to be more conservative than the average American in each year, the gap between the public's and the Court's ideological positions grows and narrows over time. We note several important events, including the backlash to the decision to overturn *Roe v. Wade* (1973) in *Dobbs v. Mississippi* (2022) that may have led the Court to swing toward the middle (5, 6), an effect that has persisted over time (7). In three of the six waves, the Court is estimated to be significantly to the right of the average citizen, whereas in the other three waves, the Court's positioning is still to the right of—but not statistically distinguishable from—the average citizen.

In addition to extending an important study, this analysis makes substantive contributions using the longer time series. We show that the Court can shift its ideological position vis-à-vis the public even when the composition of the Court remains the same. Perhaps it is unsurprising that Jessee et al. found that the court shifted to the right from 2020 to 2021 given that the location of the median voter shifted from Justice John Roberts to Justice Brett Kavanaugh after the conservatives expanded their 5–4 majority to a 6–3 majority. Yet, we find that despite the identity of the Court's ideological median not changing from 2022 to 2025, there are changes in the position of the Court relative to the public. Sometimes the Court is nearer to the position of the average Republican citizen, whereas in other years, it remains more conservative but shifts closer to the ideological position of the average American. In the conclusion, we speculate on potential mechanisms that could explain this fluctuation.

## Results

**Fig. 1.**
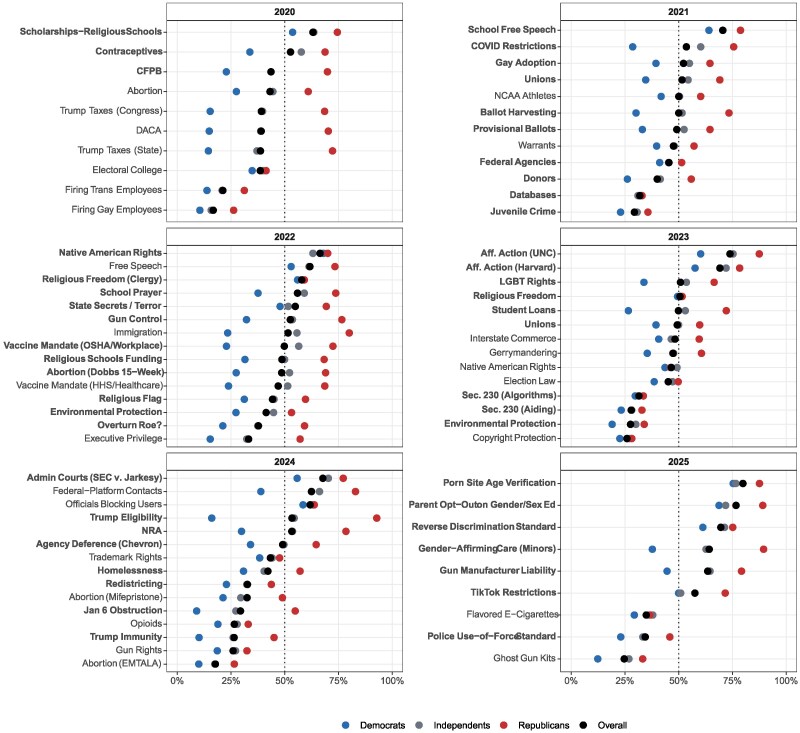
Responses to survey questions. Points show percent of respondents (or partisan subgroups) taking the conservative position on each case, defined as the position supported more by Republican respondents than by Democrats. Bolded cases indicate those where the Supreme Court's majority opinion corresponded with the conservative position under this definition.

**Fig. 2.**
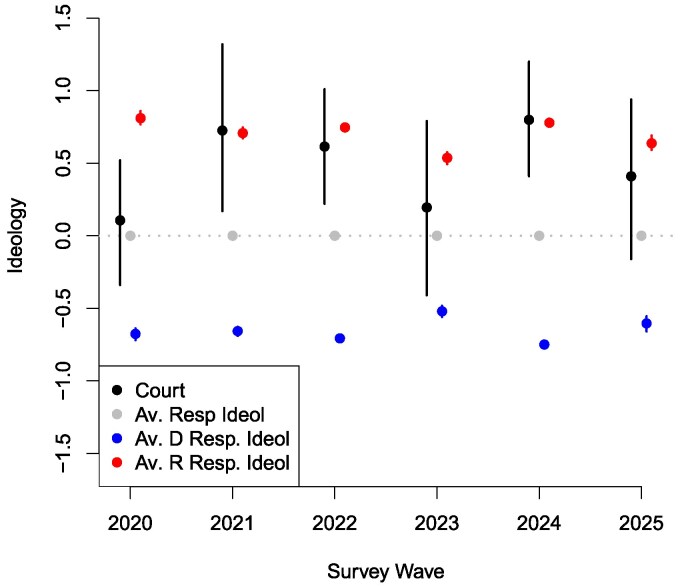
Ideological positions of the Supreme Court and the American Public, 2020–2025.

We plot respondents’ positions on the cases in Fig. 1. The main results are illustrated in Fig. 2, which plots the estimated ideological positions of four entities between 2020 and 2025: (i) the Supreme Court; (ii) the average respondent; (iii) the average Democratic respondent; and (iv) the average Republican respondent. As explained in the Materials and Methods section, these positions are estimated based on actual votes cast by the justices and survey questions administered to the public asking about their preferences on the major issues in these cases in advance of the rulings. The scale on which ideology is estimated is defined so that the location of the average respondent is 0 and the standard deviation of respondent ideologies is 1. Higher (lower) values indicate more conservative (liberal) positions, so that other estimates can be interpreted as relative to the distribution of Americans’ ideologies.

The findings from (4) are replicated in the 2020 and 2021 waves in Figure 2. The Court's position in 2020 was close to the average respondent, both substantively and significantly. However, after Justice Barrett replaced Justice Ginsburg in the fall of 2020, the Court moved to the right; and its position in the 2021 survey wave is close to the average Republican respondent and statistically distinguishable from the average respondent. The new data are presented in waves 2022–2025. In 2022, we observe a similar pattern as in 2021, with the Court located to the right of the public. This was the term in which the Court overturned *Roe v. Wade* (1973) and the ruling led to significant public backlash. Following this backlash, in 2023, our data show that the Court shifts closer to the middle, with its ideological position as close to the average respondent as it was in 2020. The 2024 and 2025 waves see further movements, with the Court shifting again to the right in 2024 (the term in which it decided *Trump v. United States*) and then back closer to the middle in 2025.

In contrast to (4), we do not find that the Court's shift to the right has increased over time. Rather, we find that the Court's ideology is generally more conservative than the public, but the size of this relative ideological gap fluctuates between 2020 and 2025, and that in some terms the Court shifts closer to the moderate position. Although we cannot know the internal motivations of the justices, it is telling that the movement closer to the middle occurred after the 2022 term, in which the Court experienced backlash from its ruling in *Dobbs v. Mississippi* (2022), and the 2024 term, in which it endured harsh criticism from its ruling in *Trump v. United States* (2024).

## Discussion

What could explain this ebb and flow of representativeness? As noted above, these fluctuations are not due to changes in the composition of the Court. The position of the Court's median voter (as reflected by the conservative 6–3 majority) has remained constant since 2021, even as Justice Ketanji Brown Jackson replaced Justice Stephen Breyer in 2022, which swapped one liberal for another. Our data cannot definitely explain these changes, but we offer conjectures. For example, one possibility is that the preferences of the Court (and the median justice specifically) became more moderate in response to *Dobbs*, perhaps because the justices were genuinely trying to better align their decisions with public opinion or strategically avoid backlash. Specifically, the Court's members may have been concerned that, if institutional legitimacy decreased, then democratically elected elites may not respect the Court's decisions or may act to curb the Court via reforms such as term limits or expanding the size of the Court (8, 9), concerns that were previously echoed by the Chief Justice (10).

However, another possibility is that the docket changed such that the Court's conservative rulings became more palatable to the public in terms following backlash. The Court sets its own docket by choosing the cases it wants to hear; it is possible for it to select cases that would allow it to maintain conservative stances on issues more in line with what the public wanted and to do so at times when avoiding additional public backlash could be important for the justices. For instance, whereas the public was generally against overturning *Roe v. Wade* (1973) (11), public opinion has generally been against race-based affirmative action (12), and most respondents in our survey (including Democratic respondents) supported the Court's decision in *Students for Fair Admission v. Harvard* (2023). In addition to the Court strategically selecting which cases it chooses to hear, litigants can also strategically bring cases in federal courts based on the perceived ideology of the Supreme Court.

## Materials and methods

### Survey methodology

We analyze data from six nationally representative surveys, each asking respondents’ opinions on the key issues in prominent cases in the Supreme Court's docket. These surveys were conducted in April 2020 (*n* = 2,000), April 2021 (*n* = 2,158), April 2022 (*n* = 2,133), April 2023 (*n* = 2,029), April 2024 (*n* = 2,218), and April 2025 (*n* = 2,201). Full question wordings (including the list of cases asked about) can be found in the Supporting Information (SI). Each survey was fielded by YouGov online using a representative sample of American adults recruited as part of their main panel study (see SI for details). Applying poststratification weights provided by YouGov ensures that the survey sample matches the target sample given nonresponse. This research received institutional review board (IRB) approval from The University of Texas at Austin (no. IRB 2020-03-0046), Stanford University (nos. IRB-55200 and IRB-18544), and Harvard University (nos. IRB21-0341 and IRB20-0407). Respondents were told that they could stop participating at any time and informed of the survey's basic content and length before agreeing to participate. Prior to starting the survey, respondents were shown an information screen that communicated participant rights, risks and benefits, and independent IRB contact information.

### Ideology estimation

Each survey wave included a set of questions asking respondents about their views related to notable cases heard by the Court in that term. Our aim was to ask respondents about the most prominent, salient cases that would be the most likely to impact public policy and, thus, also shape people's perceptions of the court. We also worked with the Supreme Court reporter at the *New York Times* to develop the list of cases, including the wording of these case questions. Our objective was not to ask respondents about cases’ jurisprudence issues, which could be meaningless to the vast majority of respondents, but, instead, to focus on the likely policy implications of the cases.

To estimate the ideological positions of justices, the Court, and survey respondents, we employ an ideal point model and estimation approach introduced by Clinton et al. (13). Letting *y_ij_* be 1 if actor *i* supports the Supreme Court's majority position on case *j* (where actor can refer to a justice or a survey respondent), the model assumes that *P*(*y_ij_* = 1) = *Ф*(*β_j_x_i_*  *−*  *α_j_*), where *β_j_* and *α_j_* are item parameters for each case and *x_i_* is respondent *i*'s ideological position. In each year, we use the justices’ votes on all cases as well as respondents’ preferences on the surveyed cases to estimate the parameters of the model. Further details about this approach, including estimation, can be found in the SI.

### Party identification

We use the question wording of the American National Election Studies to classify respondents into Republicans, Democrats, and Independents (see SI).

## Supplementary Material

pgag060_Supplementary_Data

## Data Availability

Data and replication code are available at the Harvard Dataverse: https://doi.org/10.7910/DVN/0WL3YN (14).
