# Supplementary material for: Is the Supreme Court veering rightward? The ebb and flow of representation
Source: PNAS Nexus. 2026 Mar 11;5(3):pgag060. doi: 10.1093/pnasnexus/pgag060 (PMC12997527; doi:10.1093/pnasnexus/pgag060)
Supplement: pgag060_Supplementary_Data [file pgag060_supplementary_data.pdf]

Is the Supreme Court Veering Rightward? The Ebb-and-Flow of Representation

Supporting Information (SI)

Survey Items

Half of respondents were randomly assigned to see the questions as they appear in the SI. The other half of respondents were randomly assigned to see versions of the questions with the response options reversed.

2020

Firing Gay Employees (Bostock v. Clayton County)
Question:
Some people believe that it should be illegal for employees to be fired based on their sexual orientation because it is discrimination on the basis of sex. Other people think that it should be legal because it is not discrimination on the basis of sex. What do you think?
Response options:
It should be ILLEGAL for employees to be fired based on their sexual orientation
It should be LEGAL for employers to fire people based on their sexual orientation

Firing Trans Employees (R.G. & G.R. Harris Funeral Homes v. EEOC)
Question:
Some people believe that it should be illegal for employees to be fired for being transgender because it is discrimination on the basis of sex. Other people think that it should be legal because it is not discrimination on the basis of sex. What do you think?
Response options:
It should be ILLEGAL for employees to be fired for being transgender.
It should be LEGAL for employees to be fired for being transgender.

Scholarships–Religious Schools (Espinoza v. Montana Department of Revenue)
Question:
The state of Montana has banned students from using taxpayer-subsidized scholarships to attend religious schools. Some people think this rule is an acceptable restriction. Other people think this rule violates people's constitutional rights.
What do you think?
Response options: States SHOULD be allowed to ban students from using taxpayer-subsidized scholarships to attend religious schools
States SHOULD NOT be allowed to ban students from using taxpayer-subsidized scholarships to attend religious schools

Electoral College (Chiafalo v. Washington)
Question:
In the U.S., the President is chosen by the Electoral College, comprised of "electors" from all 50 states and the District of Columbia. Some people think that states should be able to require their electors to vote for the person who won the majority of votes in the state and not some other person. However, some people think that electors should be able to vote for whomever they want. What do you think?
Response options:
States SHOULD be able to require their "electors" to vote for the candidate who won their state
States SHOULD NOT be able to require their "electors" to vote for the candidate who won their state

Trump Taxes (State) (Trump v. Vance)
Question:
New York state prosecutors are conducting a criminal investigation of President Trump. They have requested financial records related to President Trump's taxes and finances from his activities prior to becoming president from his accounting firm and other companies. Some people believe that New York state prosecutors have the right to obtain a president's tax records. Others believe that a president does not have to turn over his tax records to state prosecutors. What do you think?
Response options:
New York state prosecutors have the right to obtain a president's tax records
A president does not have to turn over his tax records to state prosecutors

DACA (Department of Homeland Security v. Regents of the University of California)
Question:
Deferred Action for Childhood Arrivals (DACA) was created by President Obama to protect undocumented immigrants who have lived in the U.S. since childhood from deportation. President Trump wants the Department of Homeland Security to end DACA. What do you think?
Response options:
DACA should remain
DACA should be ended

Trump Taxes (Congress) (Trump v. Deutsche Bank AG)
Question:
A Congressional committee has requested records related to President Trump's taxes and finances from his activities prior to becoming president from his accounting firm and other companies. Some people believe that a president should be able to block such companies from turning over his financial records to Congressional committees. Other people believe that the companies must comply with the Congressional committee's request. What do you think?
Response options:
A president SHOULD be able to block turning over his financial records to Congress
A president SHOULD NOT be able to block turning over his financial records to Congress

Abortion (June Medical Services v. Russo)
Question:
Louisiana passed a law requiring abortion providers to be able to send patients to nearby hospitals, a practice known as "admitting privileges." This law would mean that all abortion providers in the state except for one would be forced to close. Some people believe that Louisiana's law violates women's constitutional rights. Other people believe that the law does not violate women's constitutional rights. What do you think?
Response options:
States requiring abortion providers to have admitting privileges DOES violate women's constitutional rights
States requiring abortion providers to have admitting privileges DOES NOT violate women's constitutional rights

Contraceptives (Little Sisters of the Poor v. Pennsylvania)
Question:
The Affordable Care Act requires that health insurance plans for women include coverage for contraceptives (birth control), but the Trump administration recently passed regulations that greatly expanded exceptions to this mandate to include exemptions on the basis of religious or "moral" objections. Some people think that employers should not be forced to cover contraceptives if they express either a religious or a "moral" objection. Other people think that these employers should be forced to cover contraceptives. What do you think?
Response options:
Employers SHOULD NOT be forced to cover contraceptives
Employers SHOULD be forced to cover contraceptives

CFPB (Seila Law LLC v. Consumer Financial Protection Bureau)
Question:
In 2010, Congress established the Consumer Financial Protection Bureau (CFPB) as an independent consumer protection agency. In doing so Congress severely limited the President's ability to remove the agency's director. Some people think the structure of the CFPB is appropriate. Others disagree and believe that this gave the CFPB too much independent power, since it is very difficult for the President to remove the agency's director. What do you think?
Response options:
The structure of the CFPB is appropriate
The CFPB has too much independent power

<u>2021</u>

School Free Speech (Mahanoy Area School District v. B.L.)
Question:
[Group A] Some people think that public school officials can punish students for things they say or write off campus, including on social media, without violating students' First Amendment rights to free speech. Other people think that such punishments violate students' First Amendment rights to free speech. What do you think?

[Group B] Some people think that public school officials punishing students for things they say or write off campus, including on social media, violates students' First Amendment rights to free speech. Other people think that such punishments do not violate students' First Amendment rights to free speech. What do you think?

Response options:

Public school officials CAN punish students for things they say or write off campus

Public school officials CANNOT punish students for things they say or write off campus

COVID Restrictions (Roman Catholic Diocese of Brooklyn v. Cuomo)

Question:

[Group A] Many states have prohibited large in-person gatherings due to the COVID-19 pandemic. Some people think that states cannot prohibit in-person religious gatherings because of the First Amendment right to free exercise of religion. Other people think that states can prohibit in-person religious gatherings. What do you think?

[Group B] Many states have prohibited large in-person gatherings due to the COVID-19 pandemic. Some people think that states can prohibit in-person religious gatherings. Other people think that states cannot prohibit in-person religious gatherings because of the First Amendment right to free exercise of religion. What do you think?

Response options:

States CANNOT prohibit in-person religious gatherings because of the First Amendment right to free exercise of religion

States CAN prohibit in-person religious gatherings despite the First Amendment right to free exercise of religion

Warrants (Lange v. California)

Question:

[Group A] Police officers sometimes pursue people who flee in cars and who refuse to pull over. Some people think that a police officer should be able to enter someone's home without a warrant if they are in pursuit of them when that person enters their home. Other people think that it is unconstitutional for police to enter someone's home without a warrant except when there is a genuine emergency. What do you think?

[Group B] Police officers sometimes pursue people who flee in cars and who refuse to pull over. Some people think that it is unconstitutional for police to enter someone's home without a warrant except when there is a genuine emergency. Other people think that a police officer should be able to enter someone's home without a warrant if they are in pursuit of them when that person enters their home. What do you think?

Response options:

Police SHOULD be able to enter someone's house without a warrant if they are in pursuit of them when that person enters their home

Police SHOULD NOT be able to enter someone's house without a warrant if they are in pursuit of them when that person enters their home

Gay Adoption (Fulton v. City of Philadelphia)

Question:

[Group A] There are some religiously affiliated foster agencies that refuse to place foster children with same-sex couples. Some people think that governments can prohibit such agencies from participating in the foster care systems they operate unless the agencies allow children to be placed with same-sex couples. Other people think that doing so would violate the agencies' First Amendment rights to religious freedom. What do you think?
[Group B] There are some religiously affiliated foster agencies that refuse to place foster children with same-sex couples. Some people think that governments cannot prohibit such agencies from participating in the foster care systems because doing so would violate the agencies' First Amendment rights to religious freedoms. Other people think that the government can prohibit such agencies from participating in the foster care systems they operate unless the agencies allow children to be placed with same-sex couples. What do you think?
Response options:
Prohibiting religious agencies from participating in the foster care system unless they allow children to be placed with same-sex couples DOES NOT VIOLATE agencies' First Amendment rights to religious freedom
Prohibiting religious agencies from participating in the foster care system unless they allow children to be placed with same-sex couples DOES VIOLATE agencies' First Amendment rights to religious freedom

Unions (Cedar Point Nursery v. Hassid)
Question:
[Group A] California law requires that employers allow union representatives to enter a company's private property to meet with employees and solicit support for labor organizing. Some people believe that this is akin to the government taking companies' private property without compensation. Other people argue that the law is acceptable, and is not the government taking companies' private property without compensation. What do you think?
[Group B] Many states have prohibited large in-person gatherings due to the COVID-19 pandemic. Some people think that states can prohibit in-person religious gatherings. Other people think that states cannot prohibit in-person religious gatherings because of the First Amendment right to free exercise of religion. What do you think?
Response options:
States CANNOT require that employers allow union representatives to enter a company's private property
States CAN require that employers allow union representatives to enter a company's private property

Ballot Harvesting (Brnovich v. Democratic National Committee — II)
Question:
[Group A] Arizona offers in-person voting at a precinct or vote center either on election day or during an early-vote period. Many voters -- particularly racial minorities -- who vote early rely on another person to collect and drop off voted ballots. However, the Arizona legislature made it illegal to collect and deliver another person's ballot. Some people think that voters should be able to rely on another person or third party to collect and drop off ballots. Other people think that states can forbid this. What do you think?

[Group B] Arizona offers in-person voting at a precinct or vote center either on election day or during an early-vote period. Many voters -- particularly racial minorities -- who vote early rely on another person to collect and drop off voted ballots. However, the Arizona legislature made it illegal to collect and deliver another person's ballot. Some people think that states can forbid voters from relying on another person or third party to collect and drop off ballots. Other people think that voters should be able to rely on another person or third party to collect and drop off ballots. What do you think?
Response options:
Discarding entire ballots from voters who voted outside of their precinct IS LAWFUL
Discarding entire ballots from voters who voted outside of their precinct IS UNLAWFUL

NCAA Athletes (NCAA v. Alston)
Question:
[Group A] The National Collegiate Athletic Association (NCAA) strictly limits colleges and universities from providing paid compensation to college athletes. Some people think the NCAA's strict limits on paid compensation for college athletes in this manner is an unlawful form of coordination against athletes. Others disagree and think that the NCAA should be able to strictly limit colleges and universities from providing paid compensation to college athletes. What do you think?
[Group B] The National Collegiate Athletic Association (NCAA) strictly limits colleges and universities from providing paid compensation to college athletes. Some people think that the NCAA should be able to strictly limit colleges and universities from providing paid compensation to college athletes. Other people disagree and think the NCAA's strict limits on paid compensation for college athletes is an unlawful form of coordination against athletes. What do you think?
Response options:
The NCAA SHOULD NOT BE ABLE TO strictly limit paid compensation to college athletes.
The NCAA SHOULD BE ABLE TO strictly limit paid compensation to college athletes.

Provisional Ballots (Brnovich v. Democratic National Committee — I)
Question:
[Group A] In Arizona, if a voter arrives at a polling place and is not listed on the voter roll for that precinct, the voter may still cast a provisional ballot. After election day, Arizona election officials review all provisional ballots to determine the voter's identity and address. If officials determine that the voter voted outside of their precinct, the ballot is discarded in its entirety, even if the voter was eligible to vote in most of the races on the ballot. Some people believe that discarding entire ballots in this manner is unlawful. Other people believe that it is lawful. What do you think?
[Group B] In Arizona, if a voter arrives at a polling place and is not listed on the voter roll for that precinct, the voter may still cast a provisional ballot. After election day, Arizona election officials review all provisional ballots to determine the voter's identity and address. If officials determine that the voter voted outside of their precinct, the ballot is discarded in its entirety, even if the voter was eligible to vote in most of the races on the ballot. Some people believe that discarding

entire ballots in this manner is lawful. Other people believe that it is unlawful. What do you think?

Response options:

Discarding entire ballots from voters who voted outside of their precinct IS UNLAWFUL

Discarding entire ballots from voters who voted outside of their precinct IS LAWFUL

Federal Agencies (Collins v. Yellen)

Question:

[Group A] Some federal government agencies -- such as the Federal Housing Finance Agency -- are headed by a single director who may be removed from office by the president only for a specific cause rather than for any reason the president wishes. Some people think that this leadership structure is unconstitutional because it infringes on the president's authority over the executive branch, including such federal agencies. Other people disagree and think that this leadership structure is not unconstitutional because it does not infringe on the president's authority over the executive branch. What do you think?

[Group B] Some federal government agencies -- such as the Federal Housing Finance Agency -- are headed by a single director who may be removed from office by the president only for a specific cause rather than for any reason the president wishes. Some people think that this leadership structure is not unconstitutional because it does not infringe on the president's authority over the executive branch. Other people disagree and think that this leadership structure is unconstitutional because it infringes on the president's authority over the executive branch, including such federal agencies. What do you think?

Response options:

Limiting the president to only firing agency heads for a specific reason INFRINGES on the president's authority

Limiting the president to only firing agency heads for a specific reason DOES NOT INFRINGE on the president's authority

Donors (Americans for Prosperity Foundation v. Bonta)

Question:

[Group A] To detect possible fraud, the attorney general of California requires private nonprofit organizations to report the names and addresses of their major donors to the state, which keeps this information confidential. Some people think that this violates nonprofit organizations' First Amendment rights to free association because it might deter people from financially supporting them. Other people do not think that this violates nonprofit organizations' First Amendment rights to free association. What do you think?

[Group B] To detect possible fraud, the attorney general of California requires private nonprofit organizations to report the names and addresses of their major donors to the state, which keeps this information confidential. Some people do not think that this violates nonprofit organizations' First Amendment rights to free association. Other people think that this violates nonprofit organizations' First Amendment rights to free association because it might deter people from financially supporting them. What do you think?

Response options:

Requiring nonprofit organizations to report information to the state about their major donors VIOLATES their First Amendment rights
Requiring nonprofit organizations to report information to the state about their major donors DOES NOT VIOLATE their First Amendment rights

Databases (Van Buren v. United States)
Question:
[Group A] Law enforcement officers have access to several government databases to use for investigations and other law enforcement activities. Some people believe that using government databases for uses not explicitly authorized should be punished, even if the user had lawful access to the database. Others believe that using government databases for other uses not explicitly authorized should not be punished, so long as the user had lawful access to the database. What do you think?
[Group B] Law enforcement officers have access to several government databases to use for investigations and other law enforcement activities. Some people believe that using government databases for uses not explicitly authorized should not be punished, so long as the user had lawful access to the database. Others believe that using government databases for uses not explicitly authorized should be punished, even if the user had lawful access to the database. What do you think?
Response options:
Using government databases for uses not explicitly authorized SHOULD BE PUNISHED
Using government databases for uses not explicitly authorized SHOULD NOT BE PUNISHED

Juvenile Crime (Jones v. Mississippi)
Question: There are states that reserve the ability to sentence juvenile criminal defendants to life sentences without the possibility of any parole. Some people think that such juvenile defendants must be found to be incorrigible -- or impossible of being reformed -- before being sentenced to life without parole. Other people think that juveniles can be sentenced to life sentences without parole without states having to make such a determination. What do you think?
Response options: Juvenile defendants MUST BE FOUND to be incorrigible before being sentenced to a life sentence without parole; Juvenile defendants NEED NOT BE FOUND to be incorrigible before being sentenced to a life sentence without parole

<u>2022</u>

Executive Privilege (Trump v. Thompson)
Question:
[Group A] Former President Donald Trump attempted to block the release of documents concerning his role in the events of January 6 on the grounds that he has executive privilege. Some people think that executive privilege allows a former president to block the release of such records. Other people think that a former president does not have the authority to block the release of such records. What do you think?

[Group B] Former President Donald Trump attempted to block the release of documents concerning his role in the events of January 6 on the grounds that he has executive privilege. Some people think that a former president does not have the authority to block the release of such records. Other people think that executive privilege allows a former president to block the release of such records. What do you think?
Response options:
A former president CAN block the release of White House records
A former president CANNOT block the release of White House records

Native American Rights (Oklahoma v. Castro-Huerta)
Question:
[Group A] The defendant, a non-Native American, committed a crime against a Native American on Native American land. The state of Oklahoma would like to pursue criminal charges against the defendant. The defendant says that the state cannot prosecute him because the crime occurred on Native American land, and so only the federal government can prosecute him. Some people think that states cannot prosecute crimes that happen on Native American land, even if the perpetrator is non-Native American. Others think that states should be able to prosecute such cases. What do you think?
[Group B] The defendant, a non-Native American, committed a crime against a Native American on Native American land. The state of Oklahoma would like to pursue criminal charges against the defendant. The defendant says that the state cannot prosecute him because the crime occurred on Native American land, and so only the federal government can prosecute him. Some people think that states should be able to prosecute crimes that happen on Native American land if the perpetrator is non-Native American. Others think that states cannot prosecute such cases. What do you think?
Response options:
States should be able to prosecute non-Native Americans who commit crimes against Native Americans on Native American land.
States should NOT be able to prosecute non-Native Americans who commit crimes against Native Americans on Native American land.

Religious Freedom (Clergy) (Ramirez v. Collier)
Question:
[Group A] Texas law barred a death row inmate from having his pastor in the chamber during his execution and placing his hands on him while praying out loud. Some people think that barring religious clergy from entering the execution chamber and touching death row inmates violates the First Amendment protections of the free exercise of religion. Other people think that it does not. What do you think?
[Group B] Texas law barred a death row inmate from having his pastor in the chamber during his execution and placing his hands on him while praying out loud. Some people think that barring religious clergy from entering the execution chamber and touching death row inmates does not violate the First Amendment protections of the free exercise of religion. Other people think that it does. What do you think?

Response options:
Barring religious clergy from entering the execution chamber and touching death row inmates violates the First Amendment protections of the free exercise of religion.
Barring religious clergy from entering the execution chamber and touching death row inmates does NOT violate the First Amendment protections of the free exercise of religion.

School Prayer (Kennedy v. Bremerton School District)
Question:
[Group A] The football coach at a public high school led prayers with players before and after games. The school district asked him to stop and the coach refused. He was then suspended. Some people think the school district was right to suspend the coach because of the First Amendment's separation of church and state. Other people do not think the district was right to do so because of the coach's right to free exercise of religion. What do you think?
[Group B] The football coach at a public high school led prayers with players before and after games. The school district asked him to stop and the coach refused. He was then suspended. Some people think the school district was not right to suspend the coach because of the coach's free exercise of religion. Other people think the district was right to do so because of the First Amendment's separation of church and state. What do you think?
Response options:
The school district was right to suspend the coach
The school district was NOT right to suspend the coach

State Secrets / Terror (United States v. Zubaydah)
Question:
[Group A] A terrorism suspect currently being held in Guantanamo Bay says the CIA used enhaced interrogation techniques and wants it investigated. The government has declassified some information, but it claims it has a right to protect state secrets in the name of national security and is not compelled to provide evidence connected to the investigation. Some people think that the government has a right to protect state secrets in the name of national security and is not compelled to provide evidence. Other people think that the government must provide evidence in such situations. What do you think?
[Group B] A terrorism suspect currently being held in Guantanamo Bay says the CIA used enhaced interrogation techniques and wants it investigated. The government has declassified some information, but it claims it has a right to protect state secrets in the name of national security and is not compelled to provide evidence connected to the investigation. Some people think that the government must provide evidence in such situations. Other people think that the government has a right to protect state secrets in the name of national security and is not compelled to provide evidence. What do you think?
Response options:
The government has a right to protect state secrets in the name of national security and is not compelled to provide evidence.
The government must provide evidence in such situations.

Vaccine Mandate (HHS/Healthcare) (Biden v. Missouri)
Question:
[Group A] The federal Department of Health and Human Services (HHS) has issued a rule mandating that health care workers at hospitals and other facilities participating in Medicare and Medicaid be vaccinated against Covid-19 unless they qualify for religious or medical exemptions. Some people think this mandate is unlawful because it exceeds HHS's authority. Other people think this is a reasonable use of the agency's authority to ensure the safety of patients. What do you think?
[Group B] The federal Department of Health and Human Services (HHS) has issued a rule mandating that health care workers at hospitals and other facilities participating in Medicare and Medicaid be vaccinated against Covid-19 unless they qualify for religious or medical exemptions. Some people think this is a reasonable use of the agency's authority to ensure the safety of patients. Other people think this mandate is unlawful because it exceeds HHS's authority. What do you think?
Response options:
HHS's vaccination mandate is NOT lawful
HHS's vaccination mandate is lawful

Gun Control (New York State Rifle & Pistol Association v. Bruen)
Question:
[Group A] New York requires a person to show a need for self-protection in order to receive a license to carry a concealed firearm outside the home. Some people think that this law violates people's 2nd Amendment rights. Others think it does not violate people's 2nd Amendment rights. What do you think?
[Group B] New York requires a person to show a need for self-protection in order to receive a license to carry a concealed firearm outside the home. Some people think that this law does not violate people's 2nd Amendment rights. Others think that this law violates people's 2nd Amendment rights. What do you think?
Response options:
A state requiring a person to show a need for self-protection to receive a license to carry a concealed firearm outside the home violates people's 2nd Amendment rights
A state requiring a person to show a need for self-protection to receive a license to carry a concealed firearm outside the home does NOT violate people's 2nd Amendment rights

Vaccine Mandate (OSHA/Workplace) (National Federation of Independent Business v. Department of Labor)
Question:
[Group A] The federal Occupational Safety and Health Administration (OSHA) has issued a rule mandating that all employers with at least 100 employees require that their employees either be vaccinated against Covid-19 or else be tested weekly and wear masks at work. Some people think this mandate is unlawful because it exceeds OSHA's authority. Other people think this is a reasonable use of the agency's authority to protect workplace safety and health. What do you think?

[Group B] The federal Occupational Safety and Health Administration (OSHA) has issued a rule mandating that all employers with at least 100 employees require that their employees either be vaccinated against Covid-19 or else be tested weekly and wear masks at work. Some people think this is a reasonable use of the agency's authority to protect workplace safety and health. Other people think this mandate is unlawful because it exceeds OSHA's authority.
What do you think?
Response options:
OSHA's vaccination or testing mandate is NOT lawful
OSHA's vaccination or testing mandate is lawful

Religious Schools Funding (Carson v. Makin)
Question:
[Group A] The state of Maine pays private school tuition for students in rural areas that do not have public secondary schools. Maine prohibits students from using this public money to attend schools that are religious (or "sectarian"). Some people think that this is a violation of the First Amendment protections of the free exercise of religion. Other people think that this is a valid policy to maintain the separation between church and state. What do you think?
[Group B] The state of Maine pays private school tuition for students in rural areas that do not have public secondary schools. Maine prohibits students from using this public money to attend schools that are religious (or "sectarian"). Some people think that this is a valid policy to maintain the separation between church and state. Other people think that this is a violation of the First Amendment protections of the free exercise of religion. What do you think?
Response options:
Maine prohibiting state funds from being used at religious schools is a violation of the First Amendment protections of the free exercise of religion.
Maine prohibiting state funds from being used at religious schools is a valid policy to maintain the separation between church and state.

Abortion (Dobbs 15-Week) (Dobbs v. Jackson Women's Health Organization)
Question:
[Group A] A new law in Mississippi bans nearly all abortions after 15 weeks of pregnancy. Some people think that this law is unconstitutional. Others think it is constitutional. What do you think?
[Group B] A new law in Mississippi bans nearly all abortions after 15 weeks of pregnancy. Some people think that this law is constitutional. Others think it is unconstitutional. What do you think?
Response options:
Banning nearly all abortions after 15 weeks of pregnancy is unconstitutional;
Banning nearly all abortions after 15 weeks of pregnancy is constitutional

Immigration (Biden v. Texas)
Question:
[Group A] The U.S. Department of Homeland Security required noncitizens trying to reside in the U.S. to wait in Mexico while immigration officials process their cases. The Biden Administration issued an order ending this "remain in Mexico" program. In response, several states sued, saying that the Administration did not have adequate justification in ending the

program. Some people think that the Biden Administration should be able to end this program. Other people do think that the Biden Administration should not be able to do so. What do you think?
[Group B] The U.S. Department of Homeland Security required noncitizens trying to reside in the U.S. to wait in Mexico while immigration officials process their cases. The Biden Administration issued an order ending this “remain in Mexico” program. In response, several states sued, saying that the Administration did not have adequate justification in ending the program. Some people think that the Biden Administration should not be able to end this program. Other people think that the Biden Administration should be able to do so. What do you think?
Response options:
The Biden Administration should be able to end the “remain in Mexico” program
The Biden Administration should NOT be able to end the “remain in Mexico” program

Religious Flag (Shurtleff v. City of Boston)
Question:
[Group A] Upon request, the city of Boston often flies flags of different organizations in front of its city hall. The city refused to fly a religious organization’s flag bearing a Christian cross. Some people say that Boston’s refusal to fly a religious organization’s flag violated the organization’s First Amendment rights. Other people believe that it did not violate the organization’s First Amendment rights. What do you think?
[Group B] Upon request, the city of Boston often flies flags of different organizations in front of its city hall. The city refused to fly a religious organization’s flag bearing a Christian cross. Some people say that Boston’s refusal to fly a private religious organization’s flag did not violate the organization’s First Amendment rights. Other people believe that it did violate the organization’s First Amendment rights. What do you think?
Response options:
Boston’s refusal to fly a private religious organization’s flag violated the organization’s First Amendment rights.
Boston’s refusal to fly a private religious organization’s flag did NOT violate the organization’s First Amendment rights.

Environmental Protection (West Virginia v. EPA)
Question:
[Group A] Under federal law, the Environmental Protection Agency (EPA) has the authority to set emissions standards using “the best system of emission reduction.” Some people think this means that the EPA can set emissions limits on individual power plants and can also more broadly regulate emissions across the entire energy sector. Other people think that the EPA can only set limits on individual power plants but cannot more broadly regulate emissions across the entire energy sector. What do you think?
[Group B] Under federal law, the Environmental Protection Agency (EPA) has the authority to set emissions standards using “the best system of emission reduction.” Some people think that the EPA can only set limits on individual power plants but cannot more broadly regulate emissions across the entire energy sector. Other people think this means that the EPA can set

emissions limits on individual power plants and can also more broadly regulate emissions across the entire energy sector. What do you think?

Response options:

The EPA can set emissions limits on individual power plants and can also more broadly regulate emissions across the entire energy sector

The EPA can only set limits on individual power plants but cannot more broadly regulate emissions across the entire energy sector.

Free Speech (Houston Community College System v. Wilson)

Question:

[Group A] An elected member of a community college board criticized other board members and was subsequently censured (given a formal reprimand). Some people think the board violated the First Amendment rights of the elected member. Other people believe that the board did not violate the member's First Amendment rights. What do you think?

[Group B] An elected member of a community college board criticized other board members and was subsequently censured (given a formal reprimand) Some people believe that the board did not violate the member's First Amendment rights. Other people think the board violated the First Amendment rights of the elected member. What do you think?

Response options:

An elected body censuring the speech of an elected member violates the First Amendment.

An elected body censuring the speech of an elected member does NOT violate the First Amendment.

Overturn Roe? (Roe v. Wade)

Question:

Should the Supreme Court overrule Roe v. Wade, the 1973 decision that established a constitutional right to abortion and prohibited states from banning abortion before the fetus can survive outside the womb, at around 23 weeks of pregnancy?

Response options:

Yes, Roe v. Wade should be overturned

No, Roe v. Wade should NOT be overturned

2023

Copyright Protection (Andy Warhol Foundation v. Goldsmith)

Question:

[Group A] In 1981, a photographer took a photo of the musician Prince. The artist Andy Warhol made changes to this photograph and used that to create a series of famous prints. Some people think that when images are transformed like this, the original creator is not entitled to any payment. Other people think that the original creator is entitled to payment because the image is still recognizable. What do you think?

[Group B] In 1981, a photographer took a photo of the musician Prince. The artist Andy Warhol made changes to this photograph and used that to create a series of famous prints. Some

people think that the original creator is entitled to payment because the image is still recognizable. Other people think that when images are transformed like this, the original creator is not entitled to any payment. What do you think?
Response options:
The original creator is NOT entitled to payment
The original creator is entitled to payment

Aff. Action (UNC) (Students for Fair Admissions v. University of North Carolina)
Question:
[Group A] Some people think that public colleges and universities should NOT be able to use race as a factor in admissions. Other people think that they should be able to. What do you think?
[Group B] Some people think that public colleges and universities should be able to use race as a factor in admissions. Other people think that they should NOT be able to. What do you think?
Response options:
Public colleges and universities should NOT be able to use race as a factor in admissions
Public colleges and universities should be able to use race as a factor in admissions

Aff. Action (Harvard) (Students for Fair Admissions v. Harvard College)
Question:
[Group A] Some people think that private colleges and universities should NOT be able to use race as a factor in admissions. Other people think that they should be able to. What do you think?
[Group B] Some people think that private colleges and universities should be able to use race as a factor in admissions. Other people think that they should NOT be able to. What do you think?
Response options:
Private colleges and universities should NOT be able to use race as a factor in admissions
Private colleges and universities should be able to use race as a factor in admissions

Election Law (Moore v. Harper)
Question:
[Group A] Some people think that the language in the Constitution means that only state legislatures can regulate federal elections, without oversight from state courts. Other people think that state courts can exercise this oversight as they do in other areas. What do you think?
[Group B] Some people think state courts can exercise oversight over federal elections as they do in other areas. Other people think that the language in the U.S. Constitution means that only state legislatures can regulate federal elections, without oversight from state courts. What do you think?
Response options:
Only state legislatures can regulate federal elections, without oversight from state courts
State courts can exercise oversight over federal elections just like they do in other areas

Native American Rights (Haaland v. Brackeen)
Question:
[Group A] In 1978, Congress enacted a law that says that Native American children who are removed from their families should be placed with extended family members or foster homes of people who are also Native American. Some people think this law discriminates on the basis of race. Other people think it does NOT discriminate on the basis of race. What do you think?
[Group B] In 1978, Congress enacted a law that says that Native American children who are removed from their families should be placed with extended family members or foster homes of people who are also Native American. Some people think this law does NOT discriminate on the basis of race. Other people think it discriminates on the basis of race. What do you think?
Response options:
This law discriminates on the basis of race
This law does NOT discriminate on the basis of race

Gerrymandering (Merrill v. Milligan)
Question:
[Group A] One fourth of Alabama voters are Black. Alabama recently created seven Congressional districts, with only one of them being a majority-Black district. Some people think the small number of districts in which Black voters are a majority violates Section 2 of the Voting Rights Act, which bans racial discrimination in voting policies, and that the state should be forced to redraw the districts. Other people disagree and say that the original plan with one majority-Black district is lawful. What do you think?
[Group B] One fourth of Alabama voters are Black. Alabama recently created seven Congressional districts, with only one of them being a majority-Black district. Some people say that the plan with one majority-Black district is lawful. Other people think the small number of districts in which Black voters are a majority violates Section 2 of the Voting Rights Act, which bans racial discrimination in voting policies, and that the state should be forced to redraw the districts. What do you think?
Response options:
The state should be forced to redraw the district
The original plan with one majority-Black district is lawful

Interstate Commerce (National Pork Producers Council v. Ross)
Question:
[Group A] California law prohibits the sale of pork from pigs that are kept in small cages. Some people think that such a law discriminates against commerce from other states because most pork comes from outside of California. Other people think that such a law does not discriminate against commerce from other states. What do you think?
[Group B] California law prohibits the sale of pork from pigs that are kept in small cages. Some people think such a law does not discriminate against commerce from other states. Other people think that such a law discriminates against commerce from other states because most pork comes from outside of California. What do you think?
Response options:
Such law discriminates against commerce from other states

Such a law does NOT discriminate against commerce from other states

LGBT Rights (303 Creative LLC v. Elenis)
Question:
[Group A] Colorado law prohibits businesses, including creative and artistic businesses, from discriminating against gay customers. Some people think this violates business owners' rights to free speech. Other people think this does NOT violate business owners' rights to free speech. What do you think?
[Group B] Colorado law prohibits businesses, including creative and artistic businesses, from discriminating against gay customers. Some think such a law does NOT violate business owners' rights to free speech. Other people think this violates business owners' rights to free speech. What do you think?
Response options:
Such a law violates business owners' rights to free speech
Such a law does NOT violate business owners' rights to free speech

Religious Freedom (Groff v. DeJoy)
Question:
[Group A] A postal worker refused to work on Sundays for religious rea- sons. The Postal Service agreed to help him find coworkers to swap shifts with, but when he could not find any coworkers to swap with and did not show up to work multiple times, he was disciplined and then quit. Some people think that this is NOT religious discrimination. Other people think that this is religious discrimination and that the employer should have to accommodate his beliefs and should not have disciplined him. What do you think?
[Group B] A postal worker refused to work on Sundays for religious rea- sons. The Postal Service agreed to help him find coworkers to swap shifts with, but when he could not find any coworkers to swap with and did not show up to work multiple times, he was disciplined and then quit. Some people think that this is religious discrimination and that the employer should have to accommodate his beliefs and should not have disciplined him. Other people think that this is NOT religious discrimination. What do you think?
Response options:
This is NOT religious discrimination
This is religious discrimination

Student Loans (Biden v. Nebraska)
Question:
[Group A] The Biden Administration announced plans to give up to $20,000 in student loan forgiveness to people who make less than $125,000 a year. Some people think that the Biden Administration overstepped its authority with this debt forgiveness plan. Other people disagree and think that the Biden Administration did not overstep its authority. What do you think?
[Group B] The Biden Administration announced plans to give up to $20,000 in student loan forgiveness to people who make less than $125,000 a year. Some people think that the Biden Administration did not overstep its authority with this debt forgiveness plan. Other people

disagree and think that the Biden Administration overstepped its authority. What do you think?
Response options:
The Biden Administration overstepped its authority with its debt forgiveness plan
The Biden Administration did NOT overstep its authority with its debt forgiveness plan

Unions (Glacier Northwest v. International Brotherhood of Teamsters)
Question:
[Group A] Some people think that striking union workers can be held responsible for damage or loss caused to an em- ployer's property by employees stopping their work. Other people think that they cannot be held responsible because this would undermine the ability of union workers to strike. What do you think?
[Group B] Some people think that striking union workers cannot be held responsible for damage or loss caused to an employer's property by employees stopping their work because this would undermine the ability of union workers to strike. Other people think that striking union workers can be held responsible. What do you think?
Response options:
Union workers CAN be held responsible for such damage
Union workers CANNOT be held responsible for such damage

Sec. 230 (Algorithms) (Gonzalez v. Google LLC)
Question:
[Group A] Federal law states that internet companies are not responsible for hosting content that is posted by others. Some people think that large tech companies, such as Google, can be held responsible when their algorithm recommends certain content to users. Other people think that these companies cannot be held responsible for content recommended by the company's algorithms if that content was posted by other users. What do you think?
[Group B] Federal law states that internet companies are not responsible for hosting content that is posted by others. Some people think that large tech companies, such as Google, cannot be held responsible for content recommended by the company's algorithms if that content was posted by other users. Other people think that these companies can be held responsible when their algorithm recommends certain content to users. What do you think?
Response options:
Companies CAN be held responsible when their algorithm recommends content to users
Companies CANNOT be held responsible when their algorithm recommends content to users

Sec. 230 (Aiding) (Twitter v. Taamneh)
Question:
[Group A] Federal law states that social media companies are not responsible for hosting content that is posted by others. Some people think that social media companies can be held responsible for aiding and abetting terrorism for not removing content and accounts promoting terrorism. Other people think that they cannot be held responsible. What do you think?
[Group B] Federal law states that social media companies are not responsible for hosting content that is posted by others. Some people think that social media companies cannot be held responsible for aiding and abetting terrorism not removing content and accounts promoting

terrorism. Other people think that they can be held responsible. What do you think?
Response options:
Social media companies CAN be held responsible
Social media companies CANNOT be held responsible

Environmental Protection (Sackett v. EPA)
Question:
[Group A] The Clean Water Act is a federal law that prohibits the "discharge of pollutants" into "navigable waters." Some people think that this should be read broadly, to include things like wetlands. Other people think that this should be read narrowly, to include only things like streams, rivers, and lakes. What do you think?
[Group B] The Clean Water Act is a federal law that prohibits the "discharge of pollutants" into "navigable waters." Some people think that this should be read narrowly, to include only things like streams, rivers, and lakes. Other people think that this should be read broadly, to include things like wetlands. What do you think?
Response options:
The Clean Water Act should be read broadly, to include things like wetlands
The Clean Water Act should be read narrowly, to not include things like wetlands

2024

Abortion (EMTALA) (Moyle v. United States)
Question:
[Group A] Idaho law criminalizes most abortions. However, federal law requires that hospitals receiving federal funds must be able to provide abortions in medical emergencies. Some people think that Idaho law trumps federal law, and so Idaho hospitals cannot provide abortions to women in medical emergencies. Other people think that federal law trumps Idaho law, and so Idaho hospitals that receive federal funds can provide abortions. What do you think?
[Group B] Idaho law criminalizes most abortions. However, federal law requires that hospitals receiving federal funds must be able to provide abortions in medical emergencies. Some people think that federal law trumps Idaho law, and so Idaho hospitals that receive federal funds can provide abortions to women in medical emergencies. Other people think that Idaho law trumps federal law, and so Idaho hospitals cannot provide abortions. What do you think?
Response options:
Idaho hospitals CANNOT provide abortions in medical emergencies
Idaho hospitals can provide abortions in medical emergencies

Gun Rights (United States v. Rahimi)
Question:
[Group A] Some people think that barring domestic abusers from possessing firearms violates their Second Amendment right to keep and bear arms. Other people think that this does not violate their Second Amendment rights. What do you think?
[Group B] Some people think that barring domestic abusers from possessing firearms does not violate their Second Amendment right to keep and bear arms. Other people think that this does

violate their Second Amendment rights. What do you think?
Response options:
Barring domestic abusers from possessing firearms violates their Second Amendment rights
Barring domestic abusers from possessing firearms DOES NOT violate their Second Amendment rights

Opioids (Harrington v. Purdue Pharma)
Question:
[Group A]
The Sackler family, the owners of Purdue Pharma, profited for many years from the sale of opioids. They recently agreed to give billions of dollars to victims of the opioid epidemic in return for immunity from future lawsuits, but not all victims agreed to this deal. Some people think that the Sackler family receiving immunity from future lawsuits should not be allowed. Other people think this agreement should remain in place and the Sackler family should keep their immunity. What do you think?
[Group B]
The Sackler family, the owners of Purdue Pharma, profited for many years from the sale of opioids. They recently agreed to give billions of dollars to victims of the opioid epidemic in return for immunity from future lawsuits, but not all victims agreed to this deal. Some people think this agreement should remain in place and the Sackler family should keep their immunity. Other people think that the Sackler family receiving immunity from future lawsuits should not be allowed. What do you think?
Response options:
The Sackler family should keep their immunity from future lawsuits
The Sackler family should NOT keep their immunity from future lawsuits

Admin Courts (SEC v. Jarkesy)
Question:
[Group A] Some federal agencies bring actions in administrative proceedings with their own judges rather than in regular federal courts. Some people think this is unconstitutional because these proceedings do not have jury trials the way federal courts do. Other people think that such proceedings are constitutional. What do you think?
[Group B] Some federal agencies bring actions in administrative proceedings with their own judges rather than in regular federal courts. Some people think that such proceedings are constitutional. Other people think this is unconstitutional because these proceedings do not have jury trials the way federal courts do. What do you think?
Response options:
Federal agencies bringing actions in administrative proceedings rather than in federal courts is constitutional
Federal agencies bringing actions in administrative proceedings rather than in federal courts is NOT constitutional

Abortion (Mifepristone) (FDA v. Alliance for Hippocratic Medicine)
Question:
[Group A] Some people think that the Food and Drug Administration's approval of the abortion drug mifepristone (also known as RU-486), which is prescribed by a physician and taken by mouth, should be revoked. Other people think that it should not be revoked. What do you think?
[Group B] Some people think that the Food and Drug Administration's approval of the abortion drug mifepristone (also known as RU-486), which is prescribed by a physician and taken by mouth, should not be revoked. Other people think that it should be revoked. What do you think?
Response options:
The Food and Drug Administration's approval of mifepristone should be revoked
The Food and Drug Administration's approval of mifepristone should NOT be revoked

Trademark Rights (Vidal v. Elster)
Question:
[Group A] Federal law forbids trademarks that include individual names, including politicians' names. Some people think that this violates people's First Amendment right to free speech. Other people think this does not violate people's First Amendment rights.
[Group B] Federal law forbids trademarks that include individual names, including politicians' names. Some people think this does not violate people's First Amendment right to free speech. Other people think that this violates people's First Amendment rights.
Response options:
Forbing trademarks that include individual names, including politicians' names, does violate the First Amendment
Forbing trademarks that include individual names, including politicians' names, does NOT violate the First Amendment

Trump Eligibility (Trump v. Anderson)
Question:
[Group A] Some people think that Donald Trump is not eligible to run for president in 2024 because the 14th Amendment's ban on insurrectionists holding public office includes his conduct on January 6, 2021. Others disagree and think he is eligible to run. What do you think?
[Group B] Some people think that President Donald Trump is eligible to run for president in 2024. Others think that he is not eligible to run because the 14th Amendment's ban on insurrectionists holding public office includes his conduct on January 6, 2021. What do you think?
Response options:
President Donald Trump is NOT eligible to run for president in 2024
President Donald Trump is eligible to run for president in 2024

NRA (National Rifle Association v. Vullo)
Question:
[Group A] Following a school shooting, a state regulator told financial services companies that they should consider refusing to provide services to the National Rifle Association (NRA). Some

people think that the state regulator’s behavior violates the NRA’s First Amendment Rights. Other people say it does not. What do you think?
[Group B] Following a school shooting, a state regulator told financial services companies that they should consider refusing to provide services to the National Rifle Association (NRA). Some people think that the state regulator’s behavior does not violate the NRA’s First Amendment Rights. Other people say it does. What do you think?
Response options:
State regulator’s behavior DOES NOT violate the NRA’s First Amendment Rights
State regulator’s behavior violates the NRA’s First Amendment Rights

Agency Deference (Chevron) (Loper Bright v. Raimondo)
Question:
[Group A] Courts usually defer to how administrative agencies interpret laws in situations where the law is unclear. Some people think that courts should not defer to administrative agencies when laws are unclear because that gives these agencies too much power. Others think that administrative agencies employ experts, and so courts should defer to them in these situations. What do you think?
[Group B] Courts usually defer to how administrative agencies interpret laws in situations where the law is unclear. Some think that administrative agencies employ experts, and so courts should defer to administrative agencies when laws are unclear. Other people think that courts should not defer to administrative agencies in these cases because that gives these agencies too much power. What do you think?
Response options:
Courts should defer to administrative agencies when laws are unclear
Courts should NOT defer to administrative agencies when laws are unclear

Homelessness (City of Grants Pass v. Johnson)
Question:
[Group A] A local law bans homeless people from camping outside even when local shelters are full. Some people believe that the law violates the Constitution’s prohibition against cruel and unusual punishment because these people have nowhere else to go. Other people think this does not violate the Constitution’s prohibition against cruel and unusual punishment. What do you think?
[Group B] A local law bans homeless people from camping outside even when local shelters are full. Some people think that the law does not violate the Constitution’s prohibition against cruel and unusual punishment. Other people believe that this does violate the Constitution’s prohibition against cruel and unusual punishment because these people have nowhere else to go. What do you think?
Response options:
Banning homeless people from camping outside even when local shelters are full violates the Constitution
Banning homeless people from camping outside even when local shelters are full DOES NOT violate the Constitution

Redistricting (Alexander v. South Carolina State Conference of the NAACP)
Question:
[Group A] A proposed South Carolina election map moves thousands of Black voters to different districts. Some people claim the voters were moved because of their race, and so the map is unconstitutional. Other people claim the voters were not moved because of their race but because they were mostly Democrats, and so the map is constitutional. What do you think?
[Group B] A proposed South Carolina election map moves thousands of Black voters to different districts. Some people claim the voters were not moved because of their race but because they were mostly Democrats, and so the map is constitutional. Other people claim the voters were moved because of their race, and so the map is unconstitutional. What do you think?
Response options:
Moving these voters is constitutional
Moving these voters is unconstitutional

Trump Immunity (Trump v. United States)
Question:
[Group A] Some people think that former presidents are immune from criminal prosecution for actions they took while president. Other people think that former presidents are not immune from criminal prosecution for actions they took while president and so can be criminally prosecuted for such actions. What do you think?
[Group B]Some people think that former presidents are not immune from criminal prosecution for actions they took while president and so can be criminally prosecuted for such actions. Other people think that former presidents are immune from criminal prosecution for actions they took while president. What do you think?
Response options:
Former presidents are immune from criminal prosecution for actions they took while president
Former presidents are NOT immune from criminal prosecution for actions they took while president

2025

Ghost Gun Kits (Garland v. Vanderstok)
Question:
[Group A] There are kits available that allow people to make firearms at home. Some people think that the government should be able to regulate these kits just like it can regulate manufactured firearms. Other people think that the government should not be able to regulate these kits. What do you think?
[Group B] There are kits available that allow people to make firearms at home. Some people think that the government should not be able to regulate these kits. Other people think that the government should be able to regulate these kits just like it can regulate manufactured firearms. What do you think?
Response options:
The government should be able to regulate kits for making homemade firearms
The government should NOT be able to regulate kits for making homemade firearms

Gender-Affirming Care (Minors) (United States v. Skrmetti)
Question:
[Group A] Some people think that states should be able to ban certain treatments – such as puberty blockers and hormone therapy – for transgender minors. Other people think that states should not be able to ban such treatments. What do you think?
[Group B] Some people think that states should not be able to ban certain treatments – such as puberty blockers and hormone therapy – for transgender minors. Other people think that states should be able to ban such treatments. What do you think?
Response options:
States should be able to ban transgender minors from obtaining certain treatments such as puberty blockers and hormone therapy
States should NOT be able to ban transgender minors from obtaining certain treatments such as puberty blockers and hormone therapy

TikTok Restrictions (TikTok Inc. v. Garland)
Question:
[Group A] Some people think that the government should be able to ban social media platforms, such as TikTok, that are controlled by foreign adversaries. Other people think such bans violate freedom of speech and so the government should not be able to ban these platforms. What do you think?
[Group B] Some people think that the government should not be able to ban social media platforms, such as TikTok, that are controlled by foreign adversaries, because such bans violate freedom of speech. Other people think that the government should be able to ban these platforms. What do you think?
Response options:
The government should be able to ban social media platforms controlled by foreign adversaries
The government should NOT be able to ban social media platforms controlled by foreign adversaries

Flavored E-Cigarettes (FDA v. Wages and White Lion Investments, LLC)
Question:
[Group A] Some people think that the Food and Drug Administration (FDA) should be able to ban flavored e-cigarettes (or "vape" products). Other people think that it should not be able to do this. What do you think?
[Group B] Some people think that the Food and Drug Administration (FDA) should not be able to ban flavored e-cigarettes (or "vape" products). Other people think that it should be able to do this. What do you think?
Response options:
The FDA should be able to ban flavored e-cigarettes
The FDA should NOT be able to ban flavored e-cigarettes

Porn Site Age Verification (Free Speech Coalition v. Paxton)
Question:
[Group A] Some people think states should be able to require websites that publish sexual material to verify users' ages. Other people think that states should not be able to do this because it violates adults' free speech rights. What do you think?
[Group B] Some people think states should not be able to require websites that publish sexual material to verify users' ages because it violates adults' free speech rights. Other people think that states should be able to do this. What do you think?
Response options:
States should be able to require websites to verify users' ages
States should NOT be able to require websites to verify users' ages

Parent Opt-Out on Gender/Sex Ed (Mahmoud v. Taylor)
Question:
[Group A] Some public elementary schools include instruction on gender and sexuality in their curriculums. Some people think schools must give parents with religious objections the ability to opt their children out of this instruction. Other people think that schools do not need to give parents the ability to opt their children out. What do you think?
[Group B] Some public elementary schools include instruction on gender and sexuality in their curriculums. Some people think that schools do not need to give parents who have religious objections the ability to opt their children out of this instruction. Other people think that schools must give these parents the ability to opt their children out. What do you think?
Response options:
Schools must give parents who have religious objections the ability to opt out of instruction on gender and sexuality
Schools do not need to give parents who have religious objections the ability to opt out of instruction on gender and sexuality

Gun Manufacturer Liability (Smith & Wesson v. Estados Unidos Mexicanos)
Question:
[Group A] Mexican crime cartels have used firearms made in the U.S. to commit crimes in Mexico. Some people think that it should be possible to hold U.S. gun makers financially responsible for these crimes. Other people think it should not be possible. What do you think?
[Group B] Mexican crime cartels have used firearms made in the U.S. to commit crimes in Mexico. Some people think that it should not be possible to hold U.S. gun makers financially responsible for these crimes. Other people think it should be possible. What do you think?
Response options:
US gun manufacturers can be held liable for crimes committed by Mexican cartels that use their weapons
US gun manufacturers should be held liable for crimes committed by Mexican cartels that use their weapons

Reverse Discrimination Standard (Ames v. Ohio Department of Youth Services)
Question:
[Group A] Some people think that people claiming "reverse discrimination" – or discrimination against members of a majority group – should have to meet the same standards as minorities do in order to prove they have been discriminated against. Other people think that the people claiming reverse discrimination should have to show more evidence than minorities do. What do you think?
[Group B] Some people think that people claiming "reverse discrimination" – or discrimination against members of a majority group – should have to show more evidence than minorities do in order to prove they have been discriminated against. Other people think that the people claiming reverse discrimination should have to meet the same standards as minorities do. What do you think?
Response options:
People claiming "reverse discrimination" should have to meet the same standards as minorities claiming discrimination
People claiming reverse discrimination should have to show more evidence

Police Use-of-Force Standard (Barnes v. Felix)
Question:
[Group A] Police can lawfully use deadly force if their lives are in danger. Some people think that when judging whether deadly force was justified, courts should consider any actions officers took that may have unnecessarily increased the danger they faced. Other people think that only actions in the seconds just before an officer's safety was threatened matter and that earlier actions officers took that may have unnecessarily increased the danger they faced are irrelevant. What do you think?
[Group B] Police can lawfully use deadly force if their lives are in danger. Some people think that when judging whether deadly force was justified, only actions in the seconds just before an officer's safety was threatened matter and that earlier actions officers took that may have unnecessarily increased the danger they faced are irrelevant. Other people think that courts should consider any actions officers took that may have unnecessarily increased the danger they faced. What do you think?
Response options:
Courts should consider any actions officers took that may have unnecessarily increased the danger they faced
Courts should only consider actions officers took in the seconds before an officer's safety was threatened

Additional Survey Details

This research received institutional review board (IRB) approval from The University of Texas at Austin (no. IRB2020-03-0046), Stanford University (nos. IRB55200 and IRB-18544), and Harvard University (nos. IRB21-0341 and IRB20-0407).

YouGov draws a random sample of respondents from these data sources to create a target sample. It then matches individuals from its opt-in Internet survey panel via perfect replacement such that the survey sample is equivalent to the target sample. Sample matching has been shown to perform extremely well; studies that have conducted concurrent surveys comparing YouGov against probability samples demonstrate extremely similar results across sampling methods (1). This includes not only means and distributions of variables but also relationships among survey variables and similarities to real-world benchmarks. Descriptive statistics of the three samples can be found in SI Appendix, Table S4. All analyses apply poststratification weights provided by YouGov. Applying these weights ensures that the survey sample matches the target sample given nonresponse.

To measure party identification, for all survey waves, respondents were first asked "Generally speaking, do you usually think of yourself as a ..." (response options were "Republican," "Democrat," "Independent," and "Other"). Republicans and Democrats were then asked "Would you call yourself a strong [Republican/Democrat] or a not very strong [Republican/Democrat]?" Nonpartisans were asked "Do you think of yourself as closer to the Republican Party or to theDemocratic Party?" Leaners were pooled in with partisans in analyses examining effects among Republicans and Democrats.

Estimation of Ideological Positions

We estimate the ideological positions of each survey respondent, each Supreme Court justice, and the Court as a whole (along with each survey respondent's perception of the Court's position, which is not used in the present article) using the ideal point model and estimation approach of (2). Here, y_{ij} is 1 if actor *i* supports the Supreme Court's majority position on case *j* (where "actor" can be a justice, a survey respondent, or a survey respondent's perception of the Court). The probability for each response is assumed to be $P(y_{ij} = 1) = \phi(\beta_j - \alpha_j)$ where β_j is the discrimination for case *j* indicating how an individual's ideological position x_i predicts the likelihood of supporting the Court's majority position on that case, and α_j is the difficulty parameter indicating how much baseline support there is for the majority position in the case.

We estimate the parameters of this model separately using each wave (year) of our survey. To do this, we stack justices' votes on all cases in a given term, respondents' preferences on the surveyed cases, and respondents' guesses about the Court's decision in each surveyed case. Note that because our surveys do not ask respondents about most cases heard by the Court in a given term, rows for respondents (and for respondent guesses about the Court) will have mostly missing values, and will only have non-missing entries for columns corresponding to cases included in our surveys.

The model's parameters are estimated using Markov chain Monte Carlo (MCMC) methods employed in the ideal function in the pscl R package. Default arguments for priors are used (independent standard normal for each x_i and normal with mean zero and variance 25 for each of the β_j and α_j parameters). Initial values for the ideal points x_i are based on the proportion of positions taken on each case that are conservative, and initial values fo rα_j and β_j

are obtained by estimating probit regressions predicting positions for each case using these x_is. We let the sampler run for 500,000 iterations, discarding the first 100,000 iterations as “burn in” and then storing every 100th iteration thereafter, leaving us with 4,000 saved draws from the joint posterior over the model’s unknown parameters. Standard convergence diagnostics showed strong evidence of convergence.

In order to identify the scale on which ideology is estimated, we apply transformations at each iteration of the sampler to set the mean and standard deviation of respondent ideal points (ideological positions) to 0 and 1, respectively, and that higher (lower) values indicate more conservative (liberal) ideological positions. This essentially makes the ideology scale a measure of how many standard deviations more liberal or conservative a position is relative to the distribution of ideology among ordinary Americans.

References for SI

1. S. Ansolabehere, B. F. Schaffner, Does survey mode still matter? Findings from a 2010 multi-mode comparison. *Polit. Anal.* 22, 285–303 (2014).

2. J. Clinton, S. Jackman, D. Rivers, The statistical analysis of roll call data. *Am. Polit. Sci. Rev*. 98, 355–370 (2004).
